# A Study on the Current Status and Improvement of the Digital Divide among Older People in Korea

**DOI:** 10.3390/ijerph17113917

**Published:** 2020-06-01

**Authors:** Woochun Jun

**Affiliations:** Dept. of Computer Education, Seoul National University of Education, Seoul 06639, Korea; wocjun@snue.ac.kr; Tel.: +82-2-3475-2504

**Keywords:** digital divide, older people, information literacy, information utilization skills, information ethics, disabled people, farmers and fishermen, low-income people

## Abstract

In today’s knowledge- and information-based society, information literacy and information utilization skills are indicators of one’s competitiveness, and play a very important role in various fields (e.g., in one’s career, hobbies, as well as in daily life). In particular, information literacy and information utilization skills in older people are becoming essential for them to lead affluent lives. Moreover, information and communication technology is essential form of technology that can allow the elderly to ask for help in cases of emergency, as well as in daily life. Meanwhile, according to a recent Korean national statistical index, the digital divide among older people is more serious than that of the general public. The purpose of this paper is to statistically show that the digital divide among older people is more serious than other information-weak groups, as well as the general public. In addition, the purpose of this study is to identify the priorities that affect the digital divide among the three elements of the digital divide (digital access, digital capacity, and digital utilization) for older people. Based on that, we propose a variety of ways to solve the digital divide for older people. This study is expected to be widely used in future research and policies as a basis for solving the digital divide among older people.

## 1. Introduction

The 21st century is characterized by a knowledge- and information-based society, whereas literacy and utilization skills of information and communication technology are important for everyone to live their daily lives. Information and communication technology is becoming essential tool throughout all areas of everyday life, including occupation and leisure, and is the measure of individual competitiveness.

Older people are the most vulnerable people in our society; they are subjects that we need to focus on as they need help in their daily lives (e.g., for physical or economic reasons, as well as for other reasons). Information and communication technology provides older people with the necessary means to lead their daily lives. Therefore, older people also need basic information literacy and utilization skills. In this paper, information literacy means the ability to understand theory and principle of information; moreover, information utilization means the ability to solve ordinary problems in everyday life using various software tools, such as a word processor.

Older people are classified as one of the four most information-weak groups in Korea, along with disabled people, farmers, fishermen, and low-income people [1,2,3,4]. The National Information Society Agency (NIA) provides various survey and information service improvement measures to understand, as well as improve, the level of information services among people. Typical survey results include information from culture surveys, internet addiction surveys, digital divide (also called information gap) surveys, and web accessibility surveys. Among such surveys, the digital divide status surveys [1,2,3,4,5,6,7,8,9,10,11,12,13,14,15,16,17,18,19] and web accessibility surveys [20] were conducted to improve the level of information services by correctly identifying the level of information services among people.

In the various digital divide status reports, the digital divide status of four classes (older people, low-income people, disabled people, and farmers/fishermen) were reported. These reports indicate the level of digital information services among the four major classes compared to the general public, where the level of digital information services for the general public is 100. According to the reports, the digital information service level of the ‘older people’ class was reported to be the lowest among the four information-weak classes. Note that, in this paper, older people means 55 years or older. This means that the digital information level of older people needs to be greatly enhanced.

In the previous survey reports from the NIA, it was reported that older people among four information-weak groups have the lowest digital divide index. This means that the digital divide among older people can be regarded as the most serious among the four information-weak classes. Therefore, in this work, our concern is to statistically analyze the digital divide of the older people group. Specifically, the purpose of the study is to conclude, statistically, whether the digital divide among the older people group is the worst among the four classes. We are also interested in the priorities that affect the digital divide among the three elements of the digital divide (digital access, digital capacity, and digital utilization). Based on these priorities, we present reasonable measures to reduce the digital divide among older people.

The rest of this paper is organized as follows. In Section 2, we introduce related works. We discuss the introduction of the digital divide and present related research works. In Section 3, we present statistical analysis of the digital divide of four classes. In Section 4, we analyze the statistical results and present the various measures to reduce the digital divide of the older people group. Finally, in Section 5, we conclude our work and introduce further research works.

## 2. Related Works

### 2.1. Introduction of the Digital Divide

In the literature, digital divide is defined variously, as follows.

In [21], a digital divide is “any uneven distribution in the access to, use of, or impact of information and communication technologies (ICT) between any numbers of distinct groups”.

In [22], a digital divide is “a phenomenon in which the economic and social gap between those who have access to new information technology and those who do not”.

In [23], a digital divide refers to the gap between the information ownership class and the information non-ownership class. The more computers develop, the more useful the internet becomes, and the more the gap between social classes grows. In other words, the opportunity and quality of life vary depending on whether we have a good computer, whether we have a good internet connection, or know how to use the internet properly. Technological development generally enriches human life, making it more convenient, but it also tends to widen the gap between classes. New technologies are generally more likely to be monopolized by certain classes of knowledge and property, because they are expensive and complex to own and deal with.

In the Cambridge Dictionary [24], a digital divide is defined as “the problem of some members of society not having the opportunity or knowledge to use computers and the internet that others have”.

On the other hand, according to Digital Divide Council [25], a digital divide is “the gap that exists between individuals who have access to modern information and communication technology and those who lack access”.

In Korea, the National Information Society Agency (NIA) has measured digital divide status through a nationwide survey, and has announced statistical analysis results since 2002. The digital divide index, a unit of measure in the survey of digital divide status, is a quantitative and comprehensive metric that can measure and analyze gaps in information access, capacity, and utilization as a whole. In other words, the digital divide index is based on the level of information services among the general public and older people. Specifically, if the information service level of the general public is assumed to be 100, the information service level of older people to the general public was measured to calculate the gap between the general public and older people. The digital divide index has a value between 0 and 100 (points), the closer to 100, the smaller the difference, and the closer to 0, the greater the difference.

The digital divide index consists of the following specific indices. The number in parentheses represents the relative ratio to 1.

Digital Divide Index (comprehensive) = Digital Access Level (0.2) + Digital Capability Level (0.4) + Digital Utilization Level (0.4).

Access level = whether or not to have wired and wireless information devices (0.5) + whether internet access is always available (0.5).

Capacity level = PC usability (0.5) + mobile device usability (0.5).

Utilization level = whether or not to use wired and mobile internet (0.4) + diversity of internet service use (0.4) + degree of advanced internet use (0.2).

Table 1 shows the digital divide index of older people since 2014 [1,2,3,4,5,6]. Note that index is the point of older people, assuming that the digital divide index of the general public is 100.

Moreover, Table 2, Table 3 and Table 4 show access level, capacity level, and utilization level of the digital divide index older people, since 2014.

As we can see from Table 1, Table 2, Table 3 and Table 4, the overall digital divide index of older people is improving every year, but it is still lower than that of the general public. Looking at three aspects, access, capacity, and utilization, the level of access, as of 2019, is no different from that of the general public. However, capacity and utilization levels are very poor compared to the level of the general public. This can be interpreted as meaning that older people own a lot of information devices, such as smartphones, and can use those devices at any time, but in reality, they lack the ability to use information devices and do not know how to use them.

### 2.2. Previous Works

#### 2.2.1. Case of Digital Divide in Korea

There has been some research conducted on the digital divide among older people in Korea.

In [26], for example, researchers examined the relationship between the access levels to digital information services of middle-aged people and life satisfaction, and analyzed the multiple effects of online social participation activities and online network activities. The research results are as follows: first, there is a statistically significant relationship between the level of access to digital information services and life satisfaction. Second, access levels to digital information services have a significantly greater impact on life satisfaction after passing through online social participation activities and online network participation activities.

In [27], the purpose of the research is to show that information services are a democratic tool to expand the participation of senior citizens in politics—or a problem that enhances inequality in power resources. In addition, this study analyzed whether the relationship—of influence of information services and political participation—would vary, depending on generation. According to the analysis, the participation of the older generation in politics is influenced politically by information utilization, and the participation of the younger generation in politics is influenced by information access and information utilization. Moreover, the older generation failed to connect the increase in information access to the expansion of political participation, while the younger generation found that the increase in information access significantly expanded their participation in politics.

The purpose of the study in [28] is to investigate the effects of cellular phone use on the digital divide among older people. Specifically, this study examined the quality of life of older people, in relation to their understanding on how to use cellular phones, utilization of key functions of smartphones, satisfaction in life, self-respect, and self-efficiency. This study also compared the attitudes of older people and college students toward each other, pre-test and post-test. The following results were obtained. First, the analysis showed that older people have significantly improved their understanding of how to use cellular phones after education. In addition, the ability to use key functions of smartphones has improved significantly after education. Second, the quality of life of older people, such as living satisfaction, self-respect, and self-efficiency, also showed significant improvement after education compared to before. Third, the attitude of older people to the young after education, as well as the attitude toward older people to college students, changed significantly (more positively) than before education.

In [29], research work was designed to analyze the factors of the smart digital divide between people aged 65 and older, as well as seek solutions, as information services have rapidly developed due to the spread of smartphones. Six factors were derived from existing studies: self-efficiency, education, accessibility, amusement, cost rationality, and policy support for older people’s smartphones. Based on these factors, the research model was established based on the information technology acceptance model (TAM), a total of 243 senior citizens nationwide were surveyed to collect data, and the hypothesis was verified through analysis of the structural equation model (SEM). Significance has been shown in the order of policy support for smartphones, amusement, self-efficiency, accessibility, cost rationality, and education, respectively.

As society enters an aged society, the resolution of the digital divide among older people is increasingly important, but in reality, older people are receiving less attention than other classes. The study in [30] investigated various laws and regulations to suggest that library policies be considered to address the digital divide among older people; researchers also examined the guidelines and status of library services for older people. The research suggested that public libraries nationwide should be used as basic hub institutions to resolve the digital divide among senior citizens, and that services to resolve the digital divide of senior citizens at a national level should be systematically and continuously conducted through public libraries.

The study in [31] examined 1420 older people nationwide about how the information skills of older people who know how to use computers and the internet affect the satisfaction of their lives. According to the analysis results of this study, senior citizens with information capabilities are more satisfied with their lives than those without. In addition, among older people with information capabilities, life satisfaction increases as age increases, and the higher the level of education increases. Their analysis results show that there are differences in the satisfaction levels of the lives of older people with and without information capabilities—more information capabilities of older people increases life satisfaction.

#### 2.2.2. Case of Digital Divide in Other Countries

We present the previous works on the digital divide status of other countries. The following concerning status of digital divide for some countries.

In [32], the study reported inequality indexes of internet access and mobile phone ownership in order to test use of ICT goods for the digital divide in Brazil during 2005–2013. In this study, inequality indexes are specified according to main determinants based on four nationwide representative survey data from 2005 to 2013. The following results were obtained. At first, the digital divide among individuals has been decreasing fast among Brazilians year-by-year. However, there is room for policies of mass access to ICT goods, according to mobile internet broadband access. In addition, digital illiteracy by lack of education is one of the main determinants of the digital divide in Brazil, especially among old people.

The study in [33] focuses on the digital divide of old people, the so-called ‘grey divide’, among seniors over 65. In this study, based on a representative survey in Switzerland (N = 1.105), the following results were obtained. At first, it was found that internet use was strongly skewed in this group leading to a partial exclusion of the old seniors over 70. The statistical analysis shows that gender differences in internet use disappear if controlled for education, income, technical interest, pre-retirement computer use, and marital status. Moreover, it is shown that encouragement by family and friends is a strong predictor for internet use, and private learning is preferred rather than professional courses.

In [34], an online, web-based survey is used to measure the significance of computer and internet technology in the lives of adults over 60. A total of 110 individuals participated in various regions from the United States, Canada, and other countries. In addition, the 20-question survey included questions regarding individual use, opportunities for learning, family and social connectivity, and preferences for and barriers to effectual use. Based on survey results, computer and internet use are very important to the lives of old people, such as leisure, social connection, and health care, etc.

In [35], the problems of overcoming the digital divide in modern Russia among age groups were analyzed. In this work, this process is considered as one of the important factors of social support for old people. It is shown that overcoming the divide of access to digital technologies and internet accesses does not mean a solution to the digital divide problems. In other words, it is not only technical problems but also the level of socio-economic and socio-cultural relations.

The work in [36] reviewed the literature on ageism and technology impact for old people. The work also expanded the literature by discussing why ageism affected the digital divide for old people. Based on various literature works, the following factors are determinants on the digital divide for old people: lower levels of computer literacy, technophobia, lack of perceived usefulness, physical and cognitive deficits, etc.

Reducing inequality at the national level, as well as in our society, is considered one of the most important factors of well-being [37]. As discussed in literature review works on the digital divide of older people in Korea and other countries, lack of digital literacy is a common determinant affecting the digital divide of older people. It means that lack of digital literacy is the main cause of digital divide. Older people usually lack information capabilities. Information capabilities include both information literacy (basic information theories and principles) and information utilization (problem-solving ability using tools) capabilities. The lack of information capabilities can ultimately mean that there is no opportunity to learn. In other words, compared to the younger generation, the older generation lacks the opportunity to learn voluntarily and also by the government or an organization.

## 3. Statistical Analysis of Digital Divide for the Older People Group

In this section, we present a statistical analysis on the digital divide of the older people group in Korea.

### 3.1. Analysis Data and Tool

The purpose of the statistical analysis is to identify that the digital divide of the older people group is more serious than that of the other information-weak groups (disabled people, farmers/ fishermen, and low-income people). Moreover, the purpose of this statistical analysis is to identify the priorities that affect the digital divide among the three elements of the digital divide (access, capacity, and utilization).

For the analysis of this paper, we adopted nationwide statistical data from the National Information Society Agency (http://www.nia.or.kr) [1,2,3,4]. The agency has announced a digital divide status report since 2002. The report is based on nationwide samples all over Korea.

The collected data from this study were analyzed using the Statistical Package for the Social Science (SPSS) WIN 25.0 program (IBM, Armonk, NY, USA). One-way ANOVA was conducted to examine the digital divide index of the four information-weak groups, including the digital divide index of older people.

### 3.2. Digital Divide Survey Data

Digital divide status (from 2014 to 2019) based on the NIA Reports [1,2,3,4] is summarized in Table 5, Table 6 and Table 7, respectively.

As we stated earlier, the digital divide index consists of the following three specific indices.

Digital Divide Index (comprehensive) = Digital Access Level (0.2) + Digital Capability Level (0.4) + Digital Utilization Level (0.4)

As we can see from above in Table 5, Table 6, Table 7 and Table 8, overall digital divide index of older people is more serious than three other groups, although the digital divide index of older people has been increasing year-by-year. Moreover, for each of the three elements of the digital divide index (access level, capacity level, and utilization level), each digital divide index is worse than that of the other three groups.

In this research, we are interested in identifying the following facts. The first one is to identify whether the digital divide index of older people is statistically more severe than that of the other three groups. In addition, we are interested in identifying the priorities that affect the digital divide among the three elements of the digital divide (access, capacity, and utilization).

In order to achieve this purpose, we set the following two hypotheses.

**Hypothesis** **1:** *The digital divide index of older people is more severe than that of other three groups*.

**Hypothesis** **2:** *Determining the digital divide among older people is in the order of digital capacity, digital utilization, and digital access, respectively*.

### 3.3. Statistical Analysis Results

The results of the statistical analysis of the digital divide levels of the four information-weak classes are shown in Table 9.

As shown in Table 9, older people among the four information-weak groups had the lowest digital divide index, with an average of 54.62, followed by farmers and fishermen with 62.15, disabled people 67.98, and low-income people with 80.05, and statistically significant differences (F = 12.43, *p* < 0.001). Therefore, it is shown that the digital divide of older people as the most serious among the four groups.

The results of the digital divide index of older people are shown in Table 1.

As shown in Table 10, older people have the lowest average digital capacity of 38.42, followed by lower digital utilization at 53.90 and digital access at 81.48 (F = 24.76, *p* < 0.001). Thus a statistically significant difference was shown (F = 24.76, *p* < 0.001). Therefore, older people have the lowest digital capacity among three digital divide indexes, followed by digital utilization, and digital access, respectively.

## 4. The Solutions of Digital Divide for Older People

### 4.1. Analysis of Digital Divide Index of Older Peoples

In this section, we discuss reasons of low digital information service level of older people in various ways.

Among three digital divide indexes of older people, older people have the lowest digital capacity, among three digital divide indexes, followed by digital utilization, and digital access. This result can be interpreted as follows. The digital capacity level is more fundamental than the digital utilization level, and digital utilization can also be seen as dependent on digital capacity. This means that in order to utilize digital technology, older people must have digital capacity first. Therefore, let us take a closer look at the digital capacity level for the four groups.

As we mentioned earlier, digital capacity level consists of two elements, that is, PC usability and mobile device usability, respectively. PC usability is the ability to indicate whether basic activities are possible using a PC. On the other hand, mobile device usability means the ability to indicate whether basic activities are possible using mobile devices, including smartphones.

Table 11 shows seven elements of PC usability and levels of each elements for four groups. Note that each level is rated with a 4-point scale.

Table 12 shows seven elements of mobile device usability and levels of each elements for four groups. Note that each level is rated with a 4-point scale.

As we can see from Table 11 and Table 12, the lowest scores were given in all the detailed indexes of PC usability and mobile device usability for older people. Specifically, none of the detailed areas of the PC usability and the mobile device usability had a higher score than those of the other three information-weak classes. This means that the digital capacity of older people was insufficient compared to those of the other three classes.

Specifically, for PC usability, 2. Connecting and Using the internet was the highest and 6. Detection and Treatment of Malicious Code was the lowest. In addition, for mobile device usability, 1. Basic Configuration and 4. Transferring a File to Another Person were high, and 6. Detection and Treatment of Malicious Code was the lowest.

### 4.2. Improvement Plans of Digital Divide for Older People

As the previous statistical analysis shows in this work, the biggest cause of the digital divide among older people is the lack of digital capacity. Therefore, measures are needed to strengthen and address the digital capacity and digital divide of older people. According to the report [1], for involuntary non-internet users who are willing to use the internet, but are not in a condition to, the specific reasons for involuntary use are as follows:

- ‘Don’t know how to use it or it’s difficult’ (81.1%)
- ‘The internet fee is too much’ (37.1%)
- ‘No device to use’ (33.0%)
- ‘Difficult to use due to physical limitations’ (18.0%)
- ‘Not confident in solving the difficulties’ (16.9%)

Older people responded that, if they do not know well, or have problems while using digital devices, they solve the problem as follows:

- Ask for help from the family (81.9%)
- Use professional manpower (60.0%)
- Search internet information (54.1%)
- Ask an acquaintance for help (47.5%)
- Ask a friend for help (46.1%)
- Solve the problem by himself (33.9%)

For older people, the ratio of self-resolving or retrieving information was lower than that of the general public, and the rate of soliciting family help was relatively higher.

The biggest obstacle to resolving the digital divide among older people is the lack of digital capacity; in order to address this, the first step is to provide older people with opportunities to learn to improve their digital capacity.

Meanwhile, the utilization rate of e-government is increasing every year. Table 13 shows the e-government utilization rates of the general public and old people since 2014 [38]. As Table 13 shows, the utilization rate of e-government among older people is increasing gradually. E-government’s drive to strengthen and utilize its functions can help resolve the digital divide among old people.

## 5. Conclusions

In today’s modern knowledge and information-based society, the literacy and utilization of information and communication technology are a very important indicator of competitiveness for modern civilization. That is, everyone must have some level of information literacy, information utilization, or information ethic in order to lead successful lives on a daily basis. In this knowledge- and information-based society, there are those who do not receive the benefits of information and communication technologies well. Older people are the most representative of the information-weak classes, and in order to reduce the digital divide, the problem of the digital divide among older people must be solved.

Currently, the NIA operates “Learning Country” (http://www.estudy.or.kr) under its wing. Learning Country is a free online education system to improve the information service ability of the entire nation, and various information-weak classes, including old people, as well as disabled people, providing basic information skills and the ability to utilize information in their daily lives.

Educating older people with information skills is provided in various ways. Currently, software education has been mandatory in elementary, middle, and high schools since 2018, in accordance with software education operation guidelines announced by the Ministry of Education in 2015. Accordingly, software education is mainly conducted at various private institutes as well as in after-school programs. Students, as well as older people, can participate in these courses.

Each local government provides various free information education programs for senior citizens. Typically, the Seoul Metropolitan Government operates the ‘50+ Campus’ website (http://50plus.or.kr) for older people over the age of 50. The ‘50+ Campus’ provides various programs for senior citizens, including information education. Information education includes coding education, word processing, spreadsheet software, presentation software, and video editing tools. Most courses consist of four to eight weeks of curriculum.

The purpose of this study is to statistically show that the digital divide of older people among the four major information-weak classes is the most serious, and also to analyze the causes of the digital divide of older people. Specifically, we aim to show priorities on the digital divide among three elements: digital access, digital capacity, and digital utilization. For the purpose of this research, we analyzed the national statistics from NIA for the past five years. The analysis showed that the digital information level of older people was the most serious among the four information-weak classes. In addition, priorities for deciding digital divide were shown to be in the order of digital capacity, digital utilization, and digital access, respectively.

The results of this study imply the following. First, the statistical results showed that the problem of the digital divide among older people was the most serious, and also that the biggest cause of the digital divide was the lack of digital capacity. Therefore, in order to reduce the digital divide of old people in the future, it is necessary to improve digital capacity through various forms of education, etc.

Moreover, this study implies the following limitations. The use of secondary data makes it difficult to determine the cause of the in-depth digital divide, and the tool for measuring the digital divide is not new, at a time when the current PC environment is rapidly changing into a smart environment. Nevertheless, this study is meaningful in that it recognizes the seriousness of the digital divide among senior citizens in Korea, and the main contribution is that it reveals the main cause of the digital divide.

## 6. Further Research Works

The future research tasks of this study are as follows. First, we need a more detailed analysis of the digital divide among older people. In order to analyze the digital divide of older people in depth, psychological counseling data, their health information, family relationships, and income should be considered in various ways. On the other hand, with the development of information and communication technology and the emergence of smart technologies, the digital divide among older people should be examined mainly in the smart environment, not a PC-oriented environment. Therefore, it is urgent to develop standards for investigating the digital divide in smart environments.

## Figures and Tables

**Table 1:** Digital Divide Index of older people by year.

Year	Digital Divide Index
2014	42.4
2015	45.6
2016	54.0
2017	58.3
2018	63.1
2019	64.3

**Table 2:** Access Level of older people by year.

Year	Access Level
2014	67.3
2015	68.5
2016	82.5
2017	89.9
2018	90.1
2019	90.6

**Table 3:** Capacity Level of older people by year.

Year	Capacity Level
2014	23.4
2015	29.6
2016	34.9
2017	41.0
2018	50.0
2019	51.6

**Table 4:** Utilization Level of older people by year.

Year	Utilization Level
2014	39.7
2015	44.9
2016	52.2
2017	59.9
2018	62.8
2019	63.9

**Table 5:** Digital Divide Index of four groups.

	2014	2015	2016	2017	2018	2019
**Disabled people**	60.2	62.5	65.4	70.0	74.6	75.2
**Low-income People**	72.5	74.5	77.3	81.4	86.8	87.8
**Farmers and Fishermen**	51.4	55.2	61.1	64.8	69.8	70.6
**Older people**	42.4	45.6	54.0	58.3	63.1	64.3
**Average**	50.1	52.4	58.6	65.1	68.9	69.9

(Unit: %, Assume that digital divide index of the general public is 100%).

**Table 6:** Digital Access Level of four groups.

	2014	2015	2016	2017	2018	2019
**Disabled people**	79.9	83.5	88.1	91.6	92.0	92.6
**Low-income People**	82.2	87.8	89.2	94.7	94.9	95.2
**Farmers and Fishermen**	68.1	73.4	84.8	90.4	91.0	91.3
**Older people**	67.3	68.5	82.5	89.9	90.1	90.6
**Average**	72.3	73.7	84.5	91.0	91.1	91.7

(Unit: %, Assume that digital divide index of the general public is 100%).

**Table 7:** Digital Capacity Level of four groups.

	2014	2015	2016	2017	2018	2019
**Disabled people**	45.0	47.0	49.8	57.7	66.9	67.8
**Low-income People**	66.8	67.2	69.1	78.5	85.3	86.5
**Farmers and Fishermen**	40.7	41.2	46.2	53.4	63.3	63.6
**Older people**	23.4	29.6	34.9	41.0	50.0	51.6
**Average**	34.6	37.4	45.2	51.9	59.1	60.2

(Unit: %, Assume that digital divide index of the general public is 100%).

**Table 8:** Digital Utilization Level of four groups.

	2014	2015	2016	2017	2018	2019
**Disabled people**	59.7	62.4	64.6	71.5	73.6	74.0
**Low-income People**	70.3	71.5	76.9	77.7	84.3	85.4
**Farmers and Fishermen**	48.6	55.5	59.0	63.3	65.9	67.2
**Older people**	39.7	44.9	52.2	59.9	62.8	63.9
**Average**	47.7	51.6	59.0	65.3	67.7	68.8

(Unit: %, Assume that digital divide index of the general public is 100%).

**Table 9:** Summary of digital divide of four classes.

	Mean	Standard Deviation	F	*p*
**Disabled people**	67.98	6.28	12.43 ***	0.000
**Low-income People**	80.05	6.37		
**Farmers and Fishermen**	62.15	7.77		
**Older people**	54.62	9.06		
**Overall**	66.20	11.77		

*** *p* < 0.001.

**Table 10:** Summary of digital divide of older people.

	Mean	Standard Deviation	F	*p*
**Digital Access Level**	81.48	10.95	24.76 ***	0.000
**Digital Capacity Level**	38.42	11.23		
**Digital Utilization Level**	53.90	10.01		
**Overall**	57.93	20.92		

*** *p* < 0.001.

**Table 11:** Summary of PC usability of four classes.

	Disabled People	Low-income People	Farmers and Fishermen	Older People	General Public
**1. Installing and Deleting Software**	2.12	2.37	1.90	1.84	2.69
**2. Connecting and Using the internet**	2.17	2.40	1.90	1.86	2.72
**3. Web Browser Environment Settings**	1.90	2.25	1.84	1.75	2.58
**4. Connecting and Using a Variety of External Devices**	2.00	2.27	1.86	1.77	2.62
**5. Transferring Files over the internet**	2.07	2.36	1.88	1.84	2.73
**6. Detection and Treatment of Malicious Code**	1.96	2.19	1.81	1.72	2.47
**7. Creating Documents and Materials**	2.00	2.18	1.85	1.75	2.54

**Table 12:** Summary of mobile device usability of four classes.

	Disabled People	Low-income People	Farmers and Fishermen	Older People	General Public
**1. Basic Configuration**	2.51	2.89	2.43	2.42	3.06
**2. Wireless Network Settings**	2.51	2.84	2.37	2.34	3.07
**3. Move Files to a Computer**	2.28	2.53	2.16	1.98	2.83
**4. Transferring a File to Another Person**	2.49	2.89	2.45	2.42	3.07
**5. Installing and using the required apps**	2.34	2.67	2.26	2.13	2.91
**6. Detection and Treatment of Malicious Code**	2.21	2.37	2.12	1.88	2.60
**7. Creating Documents and Materials**	2.25	2.41	2.17	1.95	2.71

**Table 13:** Utilization Rate of e-Government in Korea.

	2014	2015	2016	2017	2018	2019
**General Public**	72.5	76.7	85.8	86.7	87.5	87.6
**Older people**	21.1	34.7	52.6	54.3	58.1	58.1

(Unit: %).
